# Evidence That Homeostatic Sleep Regulation Depends on Ambient Lighting Conditions during Wakefulness

**DOI:** 10.3390/clockssleep1040040

**Published:** 2019-12-11

**Authors:** Christian Cajochen, Carolin Reichert, Micheline Maire, Luc J. M. Schlangen, Christina Schmidt, Antoine U. Viola, Virginie Gabel

**Affiliations:** 1 Centre for Chronobiology, Psychiatric Hospital of the University of Basel, Wilhelm Kleinstr. 27, CH-4002 Basel, Switzerland; carolin.reichert@upk.ch; 2 Transfaculty Research Platform Molecular and Cognitive Neurosciences, University of Basel, Birmannsgasse 8, CHF-4055 Basel, Switzerland; 3 Institute of Primary Health Care (BIHAM), University of Bern, 3012 Bern, Switzerland; micheline.maire@biham.unibe.ch; 4 Intelligent Lighting Institute, School of Innovation Sciences, Department of Human Technology Interaction, Eindhoven University of Technology, 5600 MB Eindhoven, The Netherlands; l.j.m.schlangen@tue.nl; 5 GIGA-Research, Cyclotron Research Centre-In Vivo Imaging Unit, Psychology and Neuroscience of Cognition Research Unit (PsyNCog), Faculty of Psychology and Educational Sciences, University of Liège, 4000 Liège, Belgium; christina.schmidt@uliege.be; 6 PPRS-Research, 68000 Colmar, France; avi@pprs-research.com; 7 Department of Psychiatry and Behavioral Sciences, Stanford University, Palo Alto, CA 94305, USA; gabel.virginie@gmail.com

**Keywords:** circadian timing system, EEG, spectral analysis, sleepiness, melanopic equivalent daylight illuminance, melatonin, slow-wave activity

## Abstract

We examined whether ambient lighting conditions during extended wakefulness modulate the homeostatic response to sleep loss as indexed by. slow wave sleep (SWS) and electroencephalographic (EEG) slow-wave activity (SWA) in healthy young and older volunteers. Thirty-eight young and older participants underwent 40 hours of extended wakefulness [i.e., sleep deprivation (SD)] once under dim light (DL: 8 lux, 2800 K), and once under either white light (WL: 250 lux, 2800 K) or blue-enriched white light (BL: 250 lux, 9000 K) exposure. Subjective sleepiness was assessed hourly and polysomnography was quantified during the baseline night prior to the 40-h SD and during the subsequent recovery night. Both the young and older participants responded with a higher homeostatic sleep response to 40-h SD after WL and BL than after DL. This was indexed by a significantly faster intra-night accumulation of SWS and a significantly higher response in relative EEG SWA during the recovery night after WL and BL than after DL for both age groups. No significant differences were observed between the WL and BL condition for these two particular SWS and SWA measures. Subjective sleepiness ratings during the 40-h SD were significantly reduced under both WL and BL compared to DL, but were not significantly associated with markers of sleep homeostasis in both age groups. Our data indicate that not only the duration of prior wakefulness, but also the experienced illuminance during wakefulness affects homeostatic sleep regulation in humans. Thus, working extended hours under low illuminance may negatively impact subsequent sleep intensity in humans.

## 1. Introduction

It is firmly established that human sleep regulation is under the control of the circadian timing system and an hourglass process keeping track of prior sleep-wake history as conceptualized in the two-process model of sleep regulation (for a review see [1]). In fact, the amount of time spent awake prior to sleep onset is the most important determinant of sleep intensity in mammals [2]. Sleep homeostasis can be accurately tracked, quantified and modelled by electroencephalographic (EEG) slow-wave activity (SWA) during non-rapid eye movement (NREM) sleep [3], which is considered an important marker for optimal brain functioning [4]. Later studies refined the homeostatic sleep-wake process with respect to its brain topography, with frontal brain areas more susceptible in their response to prior wake duration [5,6] and also with respect to experience-dependent aspects during wakefulness. Thus, superimposed on the global homeostatic regulation of SWA, local SWA increases have been reported to depend on scheduled activity/experience such as physical activity [7], learning [8], and stress [9] volunteers or animals were exposed to prior sleep. Along these lines, Tononi and Cirelli have proposed the synaptic homeostasis hypothesis (SHY), which assumes that sleep serves to re-establish synaptic processes which have been challenged by different experiences during prior wakefulness (for a review see [10]). According to the SHY, “sleep is the price to pay for waking plasticity, to avoid runaway potentiation, decreased signal-to-noise ratio, and impaired learning due to saturation” [10].

Interestingly, the potential impact on sleep homeostatic aspects of environmental factors such as light, noise and temperature experienced during extended wakefulness have, to our best knowledge, not yet been investigated systematically under controlled laboratory conditions in humans. Light is of particular interest, since, besides its function for vision, it also activates non-image forming brain regions implicated in the regulation of circadian rhythms, mood, sleep and learning (for a review see [11]). In addition, humans living in modern societies are spending more time indoors under rather dim light conditions [12], which potentially exacerbates when working extended hours, particularly during the night. Thus, here we investigated whether different ambient lighting conditions experienced during extended wakefulness impact on sleep homeostatic regulation. The rationale for this study was twofold: First, we have evidence that evening lighting conditions modulate EEG SWA during subsequent sleep after a normal waking day [13,14,15] and second, although sleep homeostatic processes are fully operational with age, the relative SWA response to sleep loss is diminished in frontal brain areas in older compared to young healthy volunteers [16]. Thus, we hypothesized (1) that the increase in frontal EEG SWA after sleep loss is more pronounced after experienced illuminance levels at 250 lux than after experienced dim illuminance at < 8 lux in both healthy young and older volunteers, and (2) that the light induced enhancement in the EEG SWA response is stronger in young than older participants.

## 2. Results and Discussion 

**Figure 1:**
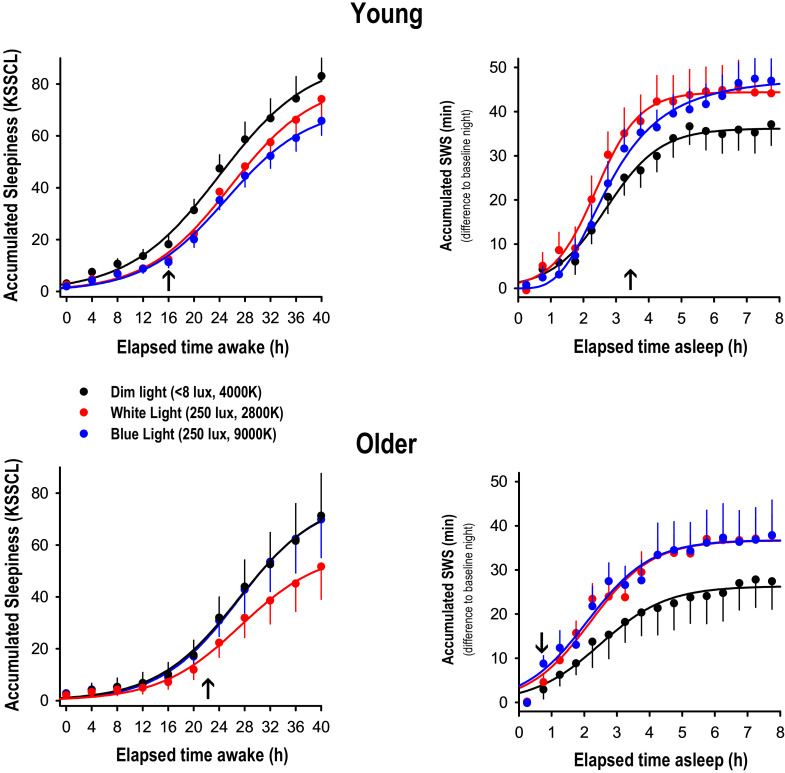
Accumulation curves of subjective sleepiness (KSSCL ratings) and SWS (min) across the 40-h SD and across the recovery night after 40-h SD under dim light (black), white light (red) and blue-enriched white light (blue); mean values + or − SEM per age group. The subjective sleepiness ratings were binned into 4-h intervals, while the SWS scores were binned into 30-min intervals. The arrows on the abscissa indicate the time point of the first occurrence of a significant difference between DL and BL or WL respectively (factor ‘light condition’, *p* at least 0.04).

As expected—confirming numerous previous reports (as an example [16,17])—sleep was more consolidated after 40-h of SD in the recovery night when compared to the baseline night as indexed by more SWS at the expense of stage 2, stage 1 and wakefulness leading to a significantly higher sleep efficiency in the recovery night (factor ‘night-type’: F_1,127_ at least 46.5, *p* at least 0.001). The response of sleep architecture to the 40-h SD was rather similar in the young and older volunteers, with the exception for stage 3 (factor ‘age’: F_1,33_ = 20.0, *p* = 0.001) and stage 4 (factor ‘age’: F_1,33_ = 16.6, *p* = 0.002) as well as rapid eye movement (REM) sleep (factor ‘age’: F_1,33_ = 4.4, *p* = 0.04). In general, older people showed a stronger increase in stage 3 sleep in response to 40-h SD, while the young, reacted with a more pronounced increase in stage 4 sleep [see relative changes in sleep architecture (recovery minus baseline night), Table 1, absolute values per baseline and recovery night are listed in the Appendix A Table A1]. This was most likely due to a reduced amplitude in sleep EEG delta waves normally occurring with healthy ageing (for a review see [18,19]). Older people responded with a slight increase in REM sleep to SD, while REM sleep in the young remained constant. A significance for the factor ‘light condition’ (F_2,50_ = 3.3, *p* = 0.04) was only found for stage 4 sleep, yielding a higher relative increase after WL and BL compared to DL in both age groups. In addition, we found a significant interaction between ‘age group’ and ‘light condition’, for sleep latency to stage 1 (F_2,51_ = 4.2, *p* = 0.02) and latency to stage 2 (F_2,51_ = 4.0, *p* = 0.02) respectively (Table 1). Unexpectedly, unlike the young volunteers, the older volunteers did not fall asleep faster after 40-SD, particularly after DL. However, this is in agreement with a previous report by Münch et al. 2004 [16], who also found a significant reduction in sleep latency after 40-SD only in the young but not older volunteers in a very similar study design. We further analyzed the intra-sleep build-up of slow wave sleep (SWS) and found a significantly faster accumulation of SWS in WL and BL compared to DL in both age groups (Figure 1, right panels). Mixed model analyses per 15-min time interval including the factors ‘age’ and ‘light condition’, yielded significance for the factor ‘light condition’ starting 3.5 h after lights off for the young, and after 75 min after lights off in the older (arrows in Figure 1, right-hand panels). The factors ‘age’ and the interaction ‘age × light condition’ did not reach significant levels in any of the time intervals. Thus, SWS accumulation during the night, a sleep homeostatic marker, was more pronounced in WL and BL than in DL in both age groups. 

**Figure 2:**
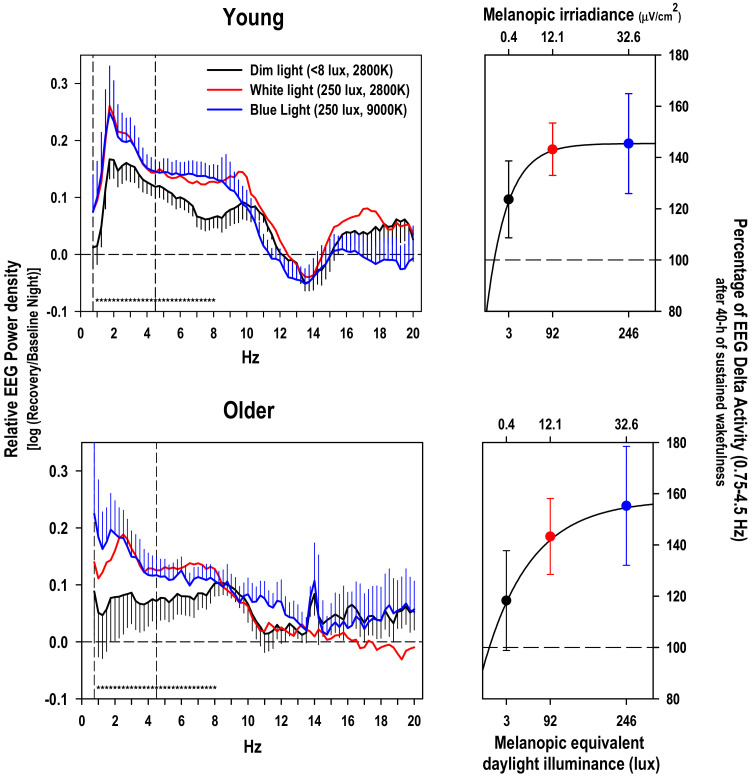
Left-hand panels: relative EEG power density during NREM sleep during the recovery night with respect to the baseline night (log ratio) in the frequency range from 0.75 to 20 Hz in the young and older group (mean values + or − SEM, black DL, red WL and in blue BL, for the sake of clarity, the SEMs for the WL condition were omitted). Stars near the abscissa indicate significant differences (*p* < 0.05) between the dim light condition and the blue and white light condition respectively (based on a significant main effect of ‘light condition’ per frequency bin over both age groups). Right-hand panels: Percentage of NREMS EEG SWA in the range of 0.75–4.5 Hz during the recovery night after 40-h SD in DL (black), WL (red), and BL (blue); mean values ± SEMs, 100% = value during the corresponding baseline night) plotted against melanopic equivalent daylight illuminance in lux, the new standard for ipRGCs driven light responses [20]. The light induced rise in EEG SWA response to 40-SD was fitted by using an exponential rise function with 3 parameters: *f* = *y*_0_ + *a* × (1 − exp(−*b* × *x*)) on the mean values.

To further corroborate our hypothesis, we calculated EEG power spectra during NREM sleep in the range of 0.75 and 20 Hz during the recovery night and expressed them relative to the corresponding values during the baseline night (Figure 2, left-hand panels).

Mixed model analyses on the relative EEG activity per single frequency bin yielded a significant effect of the factor ‘light condition’ in the range of 1 to 8 Hz (*p* at least 0.04) independent of the factor ‘EEG derivation’ and ‘age’. The factor ‘light condition’ was also significant for the collapsed frequency bins in the range from 0.75 to 4.5 Hz, the SWA band, (factor ‘light condition’, F_2,224_ = 5.72; *p* = 0.004) and the collapsed frequency bins in the theta range from 4.75 to 8 Hz, (factor ‘light condition’, F_2,224_ = 9.4; *p* = 0.0001). Post-hoc comparisons for each age group separately indicated a significantly stronger increase in relative EEG SWA after WL than after DL (*p* = 0.0005) for the young participants, while the difference between BL and DL did not reach significance. For the older group, only the difference in relative EEG SWA between BL and DL yielded significance (*p* = 0.04, Figure 2, right-hand panel). Very similar results were found for the post-hoc comparisons for the EEG theta band (data not reported).

Due to the fact that Gabel et al. 2017 [21] reported significantly lower sleepiness levels in the course of the 40-h SD under both WL and BL compared to DL in the same study, we reconfirmed this result by accumulating ratings on the Karolinska Sleepiness Symptoms Checklist (KSSCL) across the 40-h SD in both age groups (Figure 1, left-hand panel). Subjective symptoms of sleepiness were significantly reduced in both BL and WL compared to DL after 16 h of prior wakefulness in the young. In the older, only the difference between DL and WL yielded significance after 22 h of prior wakefulness. Furthermore, we tested whether these difference in subjective sleepiness were related to the observed changes in the EEG SWA response to sleep deprivation under differential lighting conditions, and did not find a significant correlation between the light induced alerting effect during wakefulness and the light-induced difference in EEG SWA response thereafter (*r* = −0.03, *p* = 0.83, Spearman rank correlation). In addition, we correlated sleepiness ratings during each 2-h time interval with relative EEG SWA and did not find consistent associations between those measures, neither a difference for either the light condition and age group (data not shown). All of this makes it unlikely that the more pronounced EEG SWA response after WL’s and BL’s was related to the alerting properties of light seen during the previous 40-h SD [21].

Based on our stringently controlled laboratory data, we have first evidence that homeostatic regulation of human sleep is modulated by prior light exposure levels, which indicates that environmental factors during wakefulness shape human sleep architecture. This goes in line with a recent sleep study performed in a nocturnal primate in the wild, reporting major influences of environmental factors (i.e., light and temperature) on monophasic sleep and activity patterns [22]. In a human field study, Wams et al. 2017 [23] found that individuals exposed to higher maximal light intensities experienced larger subsequent SWS accumulation, similarly as reported here. However, in their observational study prior wakefulness was not manipulated, and EEG delta activity was not measured. Therefore, in their study, one cannot rule out whether prior sleep had an influence on subsequent light exposure. It remains unclear whether their result reflects an effect of prior light exposure on sleep homeostasis or rather an altered circadian phase angle of entrainment. Furthermore, in their study both REM sleep and wake accumulation were reported to be affected by light exposure. In contrast, in our study we can rule out effects of sleep on subsequent light exposures and changes in circadian phase, since sleep-wake timing was controlled for. Moreover, we have no indication that circadian melatonin phase (i.e., timing of melatonin midpoint) was altered between the three light conditions [21]. This is most likely due to the fact that we exposed the study volunteers for 40 hours to light, thereby covering the phase advance and phase delay portion of the phase response curve to light in humans. Furthermore, only stage 4 sleep and EEG power density in the delta and theta range was significantly affected by the factor ‘light condition’ in our study, while REM sleep and wakefulness during sleep was not. All of this let us assume that the different lighting conditions during the 40-h SD impacted on the homeostatic aspect of sleep regulation. Although the difference in the homeostatic response to sleep deprivation between the dim and blue light condition seems rather large (21.7% increase in EEG delta activity in the young and 36.9% in the older according to Figure 2 right-hand panel), the effect size was only moderate as indexed by the Cohens’d (0.33 for the young and 0.57 for the older based on a pairwise comparison between DL and BL). Thus, the ecological validity of our laboratory study still needs to be confirmed in less controlled field studies.

We could not confirm our second hypothesis that the increase in the homeostatic sleep response by light is stronger in the young than older participants. Although, we confirmed the well-known age-related reduction in SWS and the age-related increase in wakefulness after sleep onset, the relative increase in both indices of sleep homeostasis (i.e., SWS and EEG SWA) was similarly enhanced after 40-h SD in WL and BL in both age groups. Thus, we corroborate the fact that homeostatic aspects of sleep regulation are fully operational in healthy ageing, despite lower absolute EEG SWA levels and more wakefulness after sleep onset [18,19].

Sleep alterations after exposures to differential light modalities in the evening have been frequently reported, most of them showing acute alerting responses to light extending over into the night sleep episode as verified by longer sleep latencies, reduced EEG SWA in the first cycle or REM sleep alterations [13,14,16,24]. To our best knowledge however, there is not yet a study looking at extended light exposures (i.e., 40 h) and its repercussions on subsequent sleep. Thus, although participants rated themselves less sleepy under WL and BL compared to DL in our study [21], which can be related to a tonic and less so to an acute alerting response to light, we did not find any significant association between how sleepy our participants felt during the 40-h SD and their homeostatic sleep response afterwards. This indicates that subjective sleepiness ratings, although showing an increase across the 40-SD, may not be a good predictor for homeostatic sleep regulation in the subsequent recovery night.

It is usually assumed that melatonin suppression by light is predominantly mediated via intrinsically photosensitive ganglion (ipRGCs, for a review see [25]), by activation of the photopigment melanopsin already at rather low irradiances [26,27]. If this also holds for the light induced increase in EEG SWA response to SD, one could have expected a stronger effect after BL than WL, since melanopsin excitation was a 2.7 fold stronger in BL than WL (32.6 vs. 12.1 μW/cm^2^ melanopic irradiance or 246 vs. 91.6 melanopic equivalent daylight illuminance). However, we did not find any significant differences between WL and BL in both age groups neither for stage 4 sleep, or SWS accumulation or relative EEG SWA. Thus, it could be that our observed light effects on electrophysiological correlates of sleep homeostasis were not mediated via melanopsin or that the response did already saturate out at the light level (i.e., 250 photopic lux) used in our study. Indeed, saturation of the melatonin response to polychromatic light was reported to be near to 15 μW/cm^2^ melanopic irradiance in young volunteers in the absence of a mydriatric based on the ~200 lux saturation prediction from [28] and assuming a CCT of 4000 K. Thus, at our calculated 32.6 μW/cm^2^ melanopic irradiance for the BL condition, we were probably already in the saturation part of the dose-response curve (Figure 2, right-hand panels). In other words, at light intensities of 250 photopic lux, a difference between 2800 and 9000 Kelvin does not elicit a differential response in melatonin suppression (see [21]) and EEG SWA homeostasis. In fact, the spectral composition (colour and/or correlated color temperature) of light exposure can be more important at low ambient light levels (< 200 lux): After passing a certain threshold of brightness a particular response can reach saturation, thus making the actual spectral composition above this brightness less relevant for the response-size.

Our data can be interpreted in the light of the SHY hypothesis assuming a use-dependent aspect of synaptic usage during wakefulness, which needs recovery or downscaling during subsequent sleep [10]. Along these lines, we speculate that the experienced ambient light intensity modulates “synaptic load” during wakefulness, being higher in a brighter than dim environment, and eventually leading to more EEG SWA in the subsequent night. This is in line with data in mice which demonstrated that sustained neuronal activation induced by active exploration in awake animals leads to an increase in SWA during subsequent sleep [29]. If this was true, also a more local response in EEG SWA should be expected, as demonstrated in several studies on use-dependent aspects of sleep regulation [7,8,30,31]. However, in contrast to these studies, in our study light exposure was not aimed at providing unilateral sensory stimulation and therefore did not lead to a local EEG response- at least in our 12-channel EEG recordings-, but rather led to a global EEG SWA response in all electrodes. Thus, we could not confirm our hypothesis that the increase in frontal EEG SWA after sleep loss is more pronounced in frontal brain areas after 40-h exposure to DL, WL or BL. This would rather favor the idea of a general increase in synaptic upscaling or more EEG activation, which may have been preceded by a general increase in alertness during the 40-SD.

Since specific brain regions responsible for homeostatic sleep regulation *per se* have to our knowledge not yet been assured, it is difficult to explain how light modifies sleep homeostasis on the neuroanatomical/neurophysiological level. Based on data in mice showing that melanopsin regulates both sleep-promoting and arousal-promoting responses to light [32], one could assume that ipRGC stimulation through light, particularly in the short-wavelength range, relays to the SCN and thereof to lateral hypothalamic (LH) areas for its arousal-mediating effects [33]. Conversely, light without strong short-wavelength components may decrease LH neuronal activity allowing for increased activity within the ventrolateral preoptic neurons, an important sleep-promoting area. However, how different wavelengths of light activate specific pathways for arousal promotion in humans remains elusive. Interestingly, it has been recently discovered that distinct ipRGC subpopulations mediate light’s acute effect on sleep through a circuitry distinct from that of circadian photoentrainment [34]. This corroborates how important the role of the daily cycle of light intensity is in shaping temporal sleep-activity patterns independent of circadian photoentrainment but directly via current ecological and physiological settings [35].

## 3. Methods

### 3.1. Ethical Approval 

All participants gave written informed consent for inclusion before they participated in the study. The study protocol, screening questionnaires and consent forms were approved by the local ethics committee (EKBB/Ethikkommission beider Basel, Switzerland, Project identification code: 247/11), and conformed to the Declaration of Helsinki.

### 3.2. Study Volunteers

Potential study volunteers completed a general medical questionnaire, the Epworth Sleepiness Scale (ESS), the Horne Ostberg Morningness Eveningness Questionnaire (MEQ), the Munich Chronotype Questionnaire (MCTQ), the Pittsburgh Sleep Quality Index (PSQI), and Beck Depression Inventory II (BDI-II). Based on the participants’ questionnaire data, only participants fulfilling the inclusion criteria [for details see [21]] were selected for study participation. In a next step, we ruled out sleep disturbances, and tested the volunteers’ ability to sleep in a new environment by letting them sleep one night at our Centre for Chronobiology with the entire polysomnographic (PSG) setup. In addition, each participant underwent a medical screening including an ophthalmologic examination (i.e., visual field, color vision, pupillary reflex). Female study participants took a pregnancy test and completed the study during the luteal phase of their menstrual cycle. The experimental part of the study started one week before the in-laboratory part, during which the volunteers were asked to abstain from excessive alcohol and caffeine consumption and to maintain a rather regular sleep-wake cycle (i.e., 8-h sleep at night within a regular bedtime +/− 30 min and no daytime napping) to ensure proper circadian entrainment of the sleep-wake cycle with the light-dark cycle. Compliance was verified via the use of wrist actigraphs (Actiwatch L, Cambridge Neurotechnologies, Cambridge, UK) and self-reported sleep logs. Thirty-eight healthy volunteers finally met all inclusion criteria out of an initial 650 potential participants. The young group comprised 26 participants between 20 and 35 years (11 females, 16 males, mean age (SE): 24.96 (0.58) years) and the older group included 12 participants between 55 and 75 years (3 females and 9 males, mean age (SE): 63.58 (1.27) years). For more information please refer to table 3 of [21].

### 3.3. Study Design and Light Settings

The entire in-laboratory part of the study lasted 62 h, which included a 6-h baseline evening episode, an 8-h baseline night sleep episode (BL), a 40-h total sleep deprivation (SD) followed by an 8-h recovery sleep episode (RC), all scheduled according to the individual’s usual bedtime. Each participant lived in a single windowless and sound-attenuated bedroom, which was temperature and humidity controlled without any access to time-of-day information. Visits to the bathroom were allowed via a corridor outside the bedroom under dim light conditions (<8 lux) only with blackened googles. Immediately, upon scheduled rise time from the 8-h baseline night (lights on), the light treatment started with a 40-h fluorescent white light exposure under 3 different conditions: a control dim light (DL: <8 lux, 2800 K) condition, a white light (WL: 250 lux, 2800 K) and a blue enriched white light (BL: 250 lux, 9000 K) condition. The illuminance readings were taken with a spectroradiometer (spectraval 1501, JETI Technische Instrumente GmbH, Jena/ Germany) that was oriented vertically at the eye position (and in the typical viewing direction) of the participant (for the spectral characteristics please see figure 4 of [15]). Thus, we refer here to an average illuminance incident on the eye/cornea of a volunteer in a test room with different ambient lighting conditions. The lamps in each test room were provided by Philips (Philips Lighting, Eindhoven, The Netherlands) and comprised 2700 K fluorescent tubes (Master TL5 HO 54 W/827) and 17,000 K fluorescent tubes (Master TL5 HO Activiva Active 54W 1sl). The test rooms are uniformly painted with high reflective white painting providing a homogenous light distribution. However, ambient reflections and optical conditions resulted in an effective correlated color temperature (CCT) that deviated from the values above. We effectively measured 2800 K for the DL and WL condition, and 9000 K for the BL condition. The irradiance in the DL condition was 0.0024 mW/cm^2^; photon irradiance: 6.58863 × 10^16^ photons/m^2^s, while in the WL condition: 0.07 mW/cm^2^; photon irradiance: 2.00 × 10^18^ photons/m^2^s, and in the BL condition: 0.087 mW/cm^2^; photon irradiance: 2.30 × 10^18^ photons/m^2^s. According to the new standard [20] mentioned in [36], photoreceptor weighted irradiance for melanopsin was 0.4 μW/cm^2^ (2.93 melanopic equivalent daylight illuminance) for DL, 12.1 μW/cm^2^ (91.61 melanopic equivalent daylight illuminance) and 32.6 μW/cm^2^ (246 melanopic equivalent daylight illuminance for BL. Recent evidence shows that melanopsin and/ or rhodopsin are the main drivers for melatonin suppression in humans [26,27]. Furthermore, based on a metaanalysis on a large data set from [37], Prayag et al. [38] concluded that suppression of melatonin by monochromatic lights is predominantly driven by melanopsin, and that it can be initiated at extremely low “melanopic lux” levels in experimental conditions. Here we did not calculate dose response relationships for other alpha-opic irradiances than melanopic irradiance.

Study volunteers needed to participate in at least two light conditions, while one was always the control DL condition. Out of the 26 young participants: 18 (12 m and 6 f) participated in all three lighting conditions (i.e., DL, WL, and BL): 6 with the following order: DL, BL, WL; 4 with WL, BL, DL; 3 with DL, WL, BL; 2 with WL, DL, BL; 1 with WL, DL, BL; 1 with BL, DL, WL, and 1 with BL, WL, DL. The remaining 9 (4 m and 5 f) young participated in two lighting conditions [i.e., DL and (WL or BL)]: 3 with the following order: BL, DL; 2 with DL, WL; 2 with WL, DL, and 2 with DL, WL. Out of the 12 older participants, 9 (7 m and 2 f) participated in all three lighting conditions (i.e., DL, WL, and BL): 3 with the following order: WL, DL, BL; 2 with DL, BL, WL; 1 with DL, WL, BL; 1 BL, WL, DL; 1 with WL, BL, DL, and 1 with BL, DL, WL. The remaining 3 (2 m and 1 f) older participated in two lighting conditions [i.e., DL and (WL or BL)]: 2 with the following order: DL, WL; and 1 with DL, BL. The participants were not allowed to use any light-emitting electronic devices such as smartphones, tablets or laptops during their entire stay in the laboratory. Standardized meals were provided every two hours during scheduled wakefulness and controlled for their caloric content. Participant’s movements in their room were reduced to a minimum, and they were regularly asked to take scheduled computer tests (illuminance due to screen usage <10 lx) and bathroom visits. Following activities during scheduled wakefulness during all lighting conditions were allowed: reading, listening to music, writing or drawing, knitting, doing puzzles, and talking to the study helpers. At any time, they did not have access to devices which could connect to the internet nor to other light emitting devices except for the monitor of the testing computer.

### 3.4. Polysomnographic (PSG) Recordings 

The PSG was continuously recorded during the entire 62-h stay in the laboratory. The PSG recording system (Vitaport Ambulatory system (Vitaport-3 digital recorder TEMEC Instruments BV, Kerkrade, the Netherlands) included 12 EEG derivations (Fz, F3, F4, Cz, C3, C4, Pz, P3, P4, Oz, O1, O2) referenced against linked mastoids (A1 and A2), two electrooculograms, one bipolar submental electromyogram, and one bipolar electrocardiogram. After low pass filtering all signals at 30 Hz (fourth order Bessel type anti-aliasing, total 24 dB/Oct, time constant of 1 s), online digitization with a 12 bit AD converter (0.15 μV/bit) with a sampling rate of 128 Hz for the EEG, the raw signals were stored on a flash RAM card. A single experienced sleep technician (M.F. see acknowledgments) scored the sleep stages per 20-s epochs according the standard criteria. For time course analyses we collapsed these scorings (as means per interval) into half-hourly intervals. The EEG was subjected to spectral analysis using a fast Fourier transformation (FFT; Hanning 4-s window). EEG power spectra were computed during Non-rapid eye movement (NREM) sleep in the frequency range from 0 to 20 Hz, by averaging artifact-free 4-s epochs were averaged across 20-s epochs for each EEG derivation. All PSG recordings were manually inspected for artifacts EEG channel losses etc., resulting in a total *n* = 24 for DL, *n* = 18 for WL, *n* = 19 for BL in the young, and a *n* = 12 for DL, *n* = 11 for WL, and *n* = 8 BL for the older participants.

### 3.5. Subjective Sleepiness

Subjective sleepiness was rated by the volunteers on the Karolinska sleepiness symptoms check list (KSSCL) [39] at 30-min intervals. For analyses of time courses, we collapsed these ratings into 4-hourly bins by taking the average per bin, individual, condition and night.

### 3.6. Statistical Analysis

A mixed-model analysis of variance for repeated measures (PROC MIXED, statistical package SAS [version 9.1; SAS Institute, Cary, NC, USA]) with the between factor “age” (young [Y], older [O]), and the within factors “night-type” (baseline night [BL], recovery night [RC]), and “light condition” (dim light [DL], blue-enriched white light [BL] versus white light [WL]) was calculated for the following endpoints: individual sleep variables derived from sleep scoring (Table 1), EEG power density in the frequency bins from 0.75–20 Hz and EEG slow-wave activity (SWA, EEG power density in the 0.75–4.5 Hz range). The factor “study participant” was defined as random and a compound symmetry or an autoregressive model [ar (1)] for equidistant time series was chosen as a covariance structure. The Least squares means statement was applied for *post-hoc* comparisons. Statistical differences were assumed to be significant at *p* < 0.05.

## 4. Summary and Conclusions

Our data show that the light environment impacts on human homeostatic sleep regulation. This adds to the growing insight that besides its impact on circadian physiology, light is an important environmental factor in shaping sleep-wake behavior. Although, there is evidence for a link between light exposure and subsequent sleep in the field [23], the results from this laboratory study need further validation under less controlled situations. However, they may have important ramifications when it comes to designing light solutions that support alertness or sleep promotion, while minimizing effects on the circadian timing system.

## Figures and Tables

**Table 1:** Difference in sleep variables between the recovery and baseline night in minutes for TST (total sleep time), S1 (sleep stage 1) latency, S2 (sleep stage 2) latency, and REMS (rapid eye movement sleep) latency; and in percentages of TST for Stages 1 to 4 sleep, NREMS (non-rapid eye movement sleep), REMS, wake and MT (movement time). SE (sleep efficiency) was calculated as the ratio of the duration between lights off and lights on (i.e., bedtime) and TST*100 (mean values, SEM) per light condition and age group. The last 3 columns depict significances for the factors ‘light condition’, ‘age’ and their interaction term assessed via the mixed linear model.

Age Group	Young			Older			Light Condition	Age	Light Condition × Age
***N***	24	18	17	10	10	8			
**Light condition**	Dim Light	White Light	Blue Light	Dim Light	White Light	Blue Light			
**TST (min)**	26.2 ± 6	30.8 ± 8.9	33.7 ± 9.9	12.9 ± 5.1	36.6 ± 15.2	26.6 ± 9.6	n.s.	n.s.	n.s.
**SE (%)**	3 ± 0.7	5.3 ± 2	4.2 ± 1.2	3 ± 1	6.5 ± 2.7	5.8 ± 1.7	0.08	n.s.	n.s.
**MT (%)**	−0.1 ± 0.1	0 ± 0.1	−0.2 ± 0.2	0.3 ± 0.1	0.4 ± 0.1	0.3 ± 0.2	n.s.	*	n.s.
**Wake (%)**	−3.4 ± 0.8	−7.2 ± 3	−5.2 ± 1.6	−4.8 ± 1.4	−10.2 ± 5	−8.8 ± 2.5	0.08	n.s.	n.s.
**Stage 1 (%)**	−3.2 ± 0.6	−2.5 ± 0.8	−3 ± 0.8	−2.2 ± 0.8	−4.5 ± 1.4	−3.9 ± 1.9	n.s.	n.s.	n.s.
**Stage 2 (%)**	−4.5 ± 0.9	−5.2 ± 1.4	−5.5 ± 1.1	−6.5 ± 2.3	−6.2 ± 2.6	−7.6 ± 2.2	n.s.	n.s.	n.s.
**Stage 3 (%)**	0.6 ± 0.7	1.9 ± 1	0.2 ± 0.7	5 ± 1.4	6.1 ± 1.1	4.8 ± 1.3	n.s.	*	n.s.
**Stage 4 (%)**	6.9 ± 0.7	6.7 ± 0.8	8.8 ± 0.7	2.7 ± 0.9	2.8 ± 0.8	4 ± 2	*	*	n.s.
**SWS (%)**	7.5 ± 1	8.6 ± 1.3	8.9 ± 0.8	7.7 ± 1.7	8.9 ± 1.3	8.9 ± 1.7	n.s.	n.s.	n.s.
**NREMS (%)**	3 ± 1	3.4 ± 1.4	3.5 ± 1.1	1.1 ± 1.1	2.8 ± 1.8	1.2 ± 2.1	n.s.	n.s.	n.s.
**REMS (%)**	0.2 ± 0.7	−1 ± 1.2	−0.5 ± 0.8	1 ± 1.3	1.7 ± 1.9	2.7 ± 1.3	n.s.	*	n.s.
**S1 Latency (min)**	−3.9 ± 4.5	0.1 ± 2.6	3.4 ± 2.2	8.3 ± 2.2	0.3 ± 5.8	5.4 ± 3.5	n.s.	n.s.	*
**S2 Latency (min)**	−0.4 ± 2.6	−0.7 ± 2.8	3 ± 2.3	10 ± 2.3	3.7 ± 3.9	4.8 ± 3.2	0.07	n.s.	*
**REMS Latency (min)**	−39.7 ± 8.6	−30.1 ± 16.1	−28.7 ± 9.9	−14.8 ± 6.8	−9.8 ± 16.3	−13 ± 6.6	n.s.	n.s.	n.s.

* *p* < 0.05; n.s. = not significant, see methods for more information.

## Appendix A

**Table A1:** Sleep variables during the baseline and recovery night in minutes for TST (total sleep time), S1 (sleep stage 1) latency, S2 (sleep stage 2) latency, and REMS (rapid eye movement sleep) latency; and in percentages of TST for Stages 1 to 4 sleep, NREMS (non-rapid eye movement sleep), REMS, wake and MT (movement time). SE (sleep efficiency) was calculated as the ratio of the duration between lights off and lights on (i.e., bedtime) and TST*100 (mean values) per light condition and age group. For statistics see text and Table 1.

Age Group	Young						Older					
**Light condition**	Dim Light	Dim Light	White Light	White Light	Blue Light	Blue Light	Dim Light	Dim Light	White Light	White Light	Blue Light	Blue Light
**Night**	Baseline	Recovery	Baseline	Recovery	Baseline	Recovery	Baseline	Recovery	Baseline	Recovery	Baseline	Recovery
***N***	24	24	18	18	17	17	10	10	10	10	8	8
**TST (min)**	439.6	465.8	425.7	456.5	430.7	464.4	391.9	404.8	369.0	405.6	381.0	407.7
**SE (%)**	91.3	94.2	88.4	93.7	89.8	94.0	81.7	84.8	76.9	83.4	79.3	85.1
**MT (%)**	0.9	0.8	0.9	0.9	1.0	0.8	0.3	0.7	0.4	0.8	0.3	0.7
**Wake (%)**	8.7	5.4	13.2	6.0	10.9	5.7	22.5	17.7	30.6	20.5	25.8	17.1
**Stage 1 (%)**	9.7	6.5	9.3	6.9	10.3	7.2	11.4	9.2	13.5	9.0	13.7	9.8
**Stage 2 (%)**	47.5	43.0	48.2	43.0	48.8	43.3	59.4	52.8	61.0	54.8	60.0	52.4
**Stage 3 (%)**	11.4	12.0	10.6	12.5	10.6	10.8	8.8	13.9	6.5	12.6	5.5	10.3
**Stage 4 (%)**	10.9	17.8	10.4	17.2	10.2	19.0	1.3	4.0	0.9	3.8	1.0	5.0
**SWS (%)**	22.3	29.8	21.1	29.7	20.9	29.8	10.2	17.8	7.5	16.4	6.4	15.3
**NREMS (%)**	69.8	72.7	69.3	72.7	69.6	73.1	69.5	70.7	68.5	71.2	66.5	67.7
**REMS (%)**	20.3	20.5	21.1	20.2	19.9	19.4	18.8	19.9	17.8	19.5	19.6	22.2
**S1 Latency (min)**	12.9	8.9	10.8	10.9	7.8	11.2	9.7	18.0	16.8	17.1	12.6	18.0
**S2 Latency (min)**	11.2	10.8	13.6	12.9	10.7	13.7	10.8	20.7	15.1	18.8	16.3	21.1
**REMS Latency (min)**	107.4	67.8	125.4	94.5	107.0	78.3	79.7	64.9	79.7	69.9	80.8	67.8
